# Supplementary material for: Regulatory Approved Monoclonal Antibodies Contain Framework Mutations Predicted From Human Antibody Repertoires
Source: Front Immunol. 2021 Sep 27;12:728694. doi: 10.3389/fimmu.2021.728694 (PMC8503325; doi:10.3389/fimmu.2021.728694)
Supplement: Supplementary file 1 [file DataSheet_1.pdf]

Supplementary Material

Regulatory approved monoclonal antibodies contain framework mutations predicted from human antibody repertoires

Brian M. Petersen[1], Sophia A. Ulmer[1], Emily R. Rhodes[1], Matias F Gutierrez Gonzalez[2], Brandon J Dekosky[2,3], Kayla G Sprenger[1,*], Timothy A. Whitehead[1,*]

[1]Department of Chemical and Biological Engineering, University of Colorado, Boulder, CO 80305, USA

[2]Department Pharmaceutical Chemistry, University of Kansas, Lawrence, KS 66047, USA

[3]Department of Chemical Engineering, University of Kansas, Lawrence, KS 66047, USA

***Correspondence:**
Timothy A. Whitehead: timothy.whitehead@colorado.edu

Kayla G. Sprenger: Kayla.Sprenger@colorado.edu

JSC Biotechnology Building

3415 Colorado Avenue, Boulder, CO 80305

1 Supplementary Data

Data S1. PSSMs for each of the 25 analyzed germline genes in csv format.

Data S2. FR scores and number of mutations for 1,000 randomly sampled repertoire antibody sequences across all germlines.

2 Supplementary Figures and Tables

2.1 Supplementary Figures

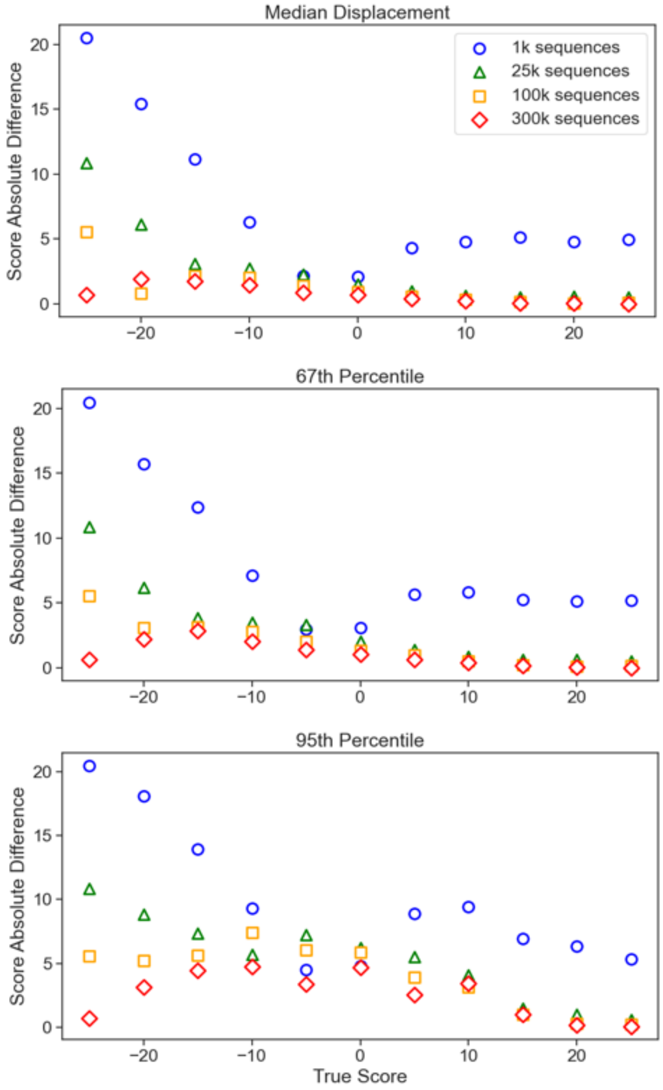

Figure S1 | Random subsampling analysis. Simple random samples without replacement of VH5-51 repertoire sequences were pulled from the dataset in the above quantities (300,000; 100,000; 25,000; 1,000). Each sample was run through a multiple sequence alignment and a PSSM was generated. Absolute differences between the score using the full number of sequences available and subsample scores were calculated and binned. For each bin, the median, 67th percentile (1σ), and 95th percentile (2σ) values were calculated and plotted. A cutoff was set at 100,000 sequences as the 25,000 and 1,000 sequences resulted in a significant increase in variability, especially at the 95th percentile.

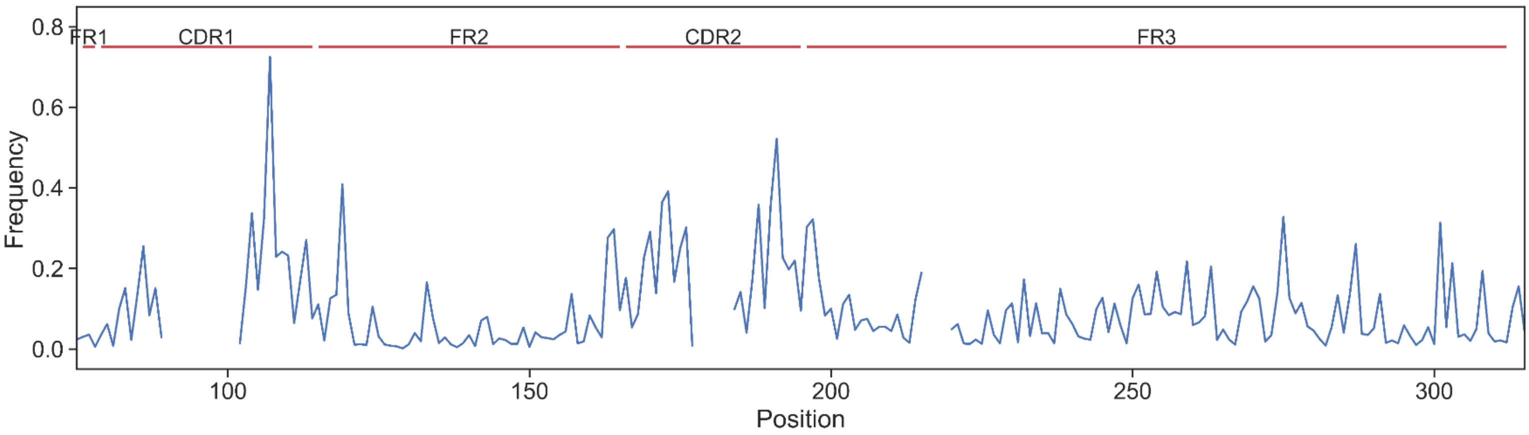

Figure S2 | Mutation frequency for all IgG sequences by position.

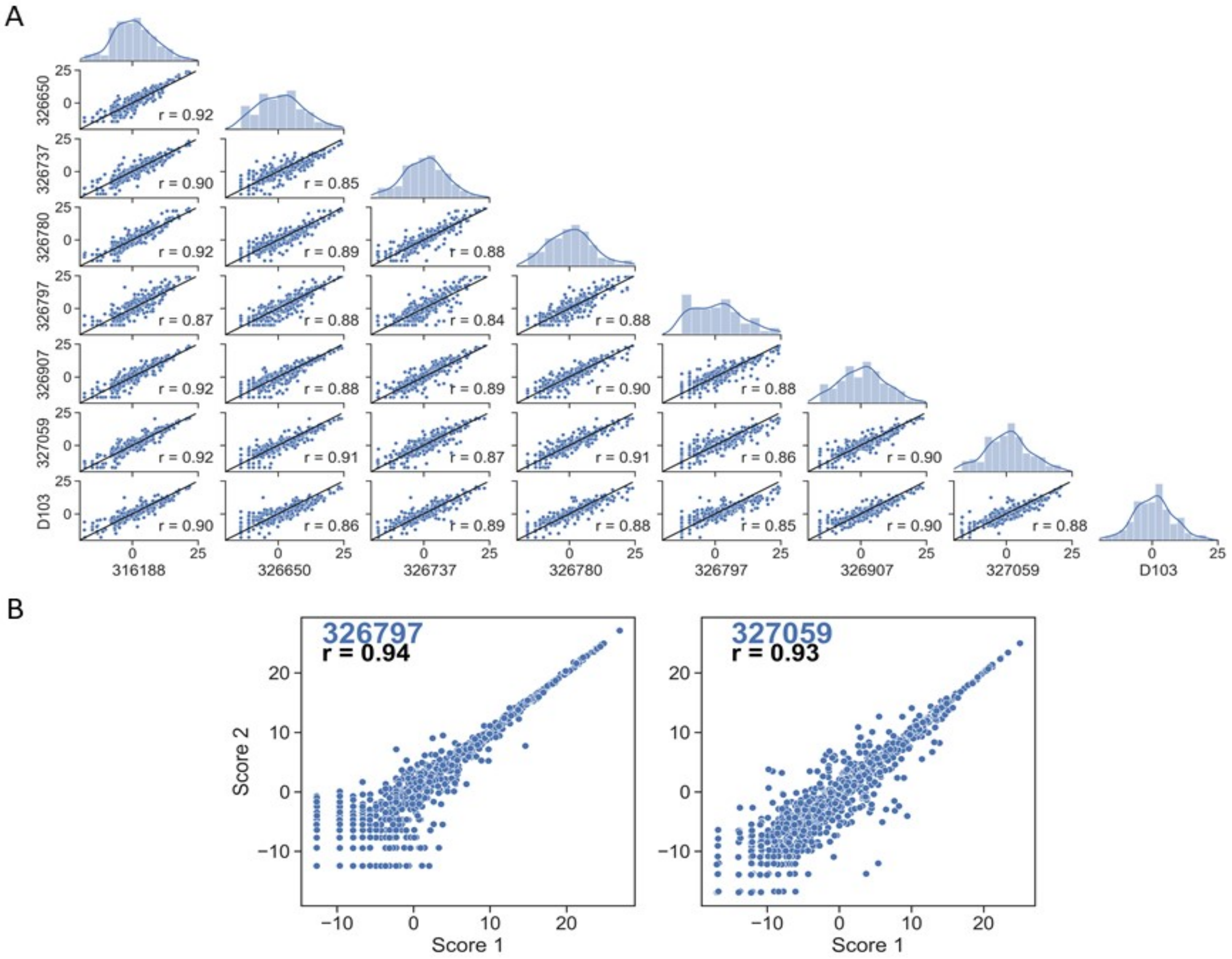

Figure S3 | Score correlations for patient-specific PSSMs. (A) Pairwise correlations between scores of patient-specific PSSMs for mutations that are a single nucleotide away from the germline codon. (B) Basal noise for two patients using two independent random samples of 50,000 sequences to generate patient-specific PSSMs. Pearson's correlation coefficients (r) are calculated for comparisons. Alphanumeric characters on the y- and x-axes represent de-identified subjects.

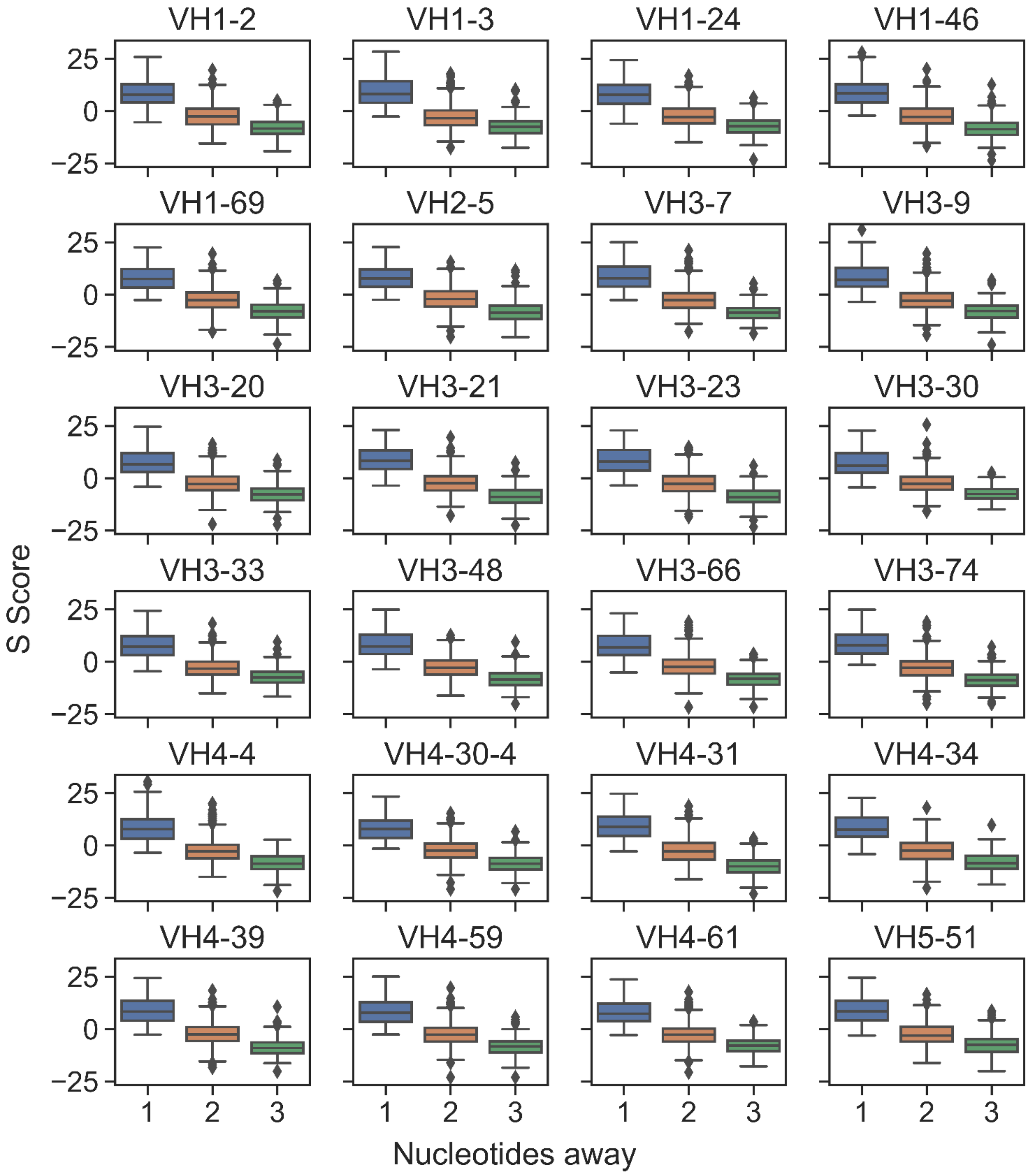

Figure S4 | Mutation scores by nucleotide distance from germline. S scores are grouped by minimum number of nucleotide changes from germline to produce the mutation (Blue: 1-nt; Orange: 2-nt; Green: 3-nt). Positions which include allelic variations are not included.

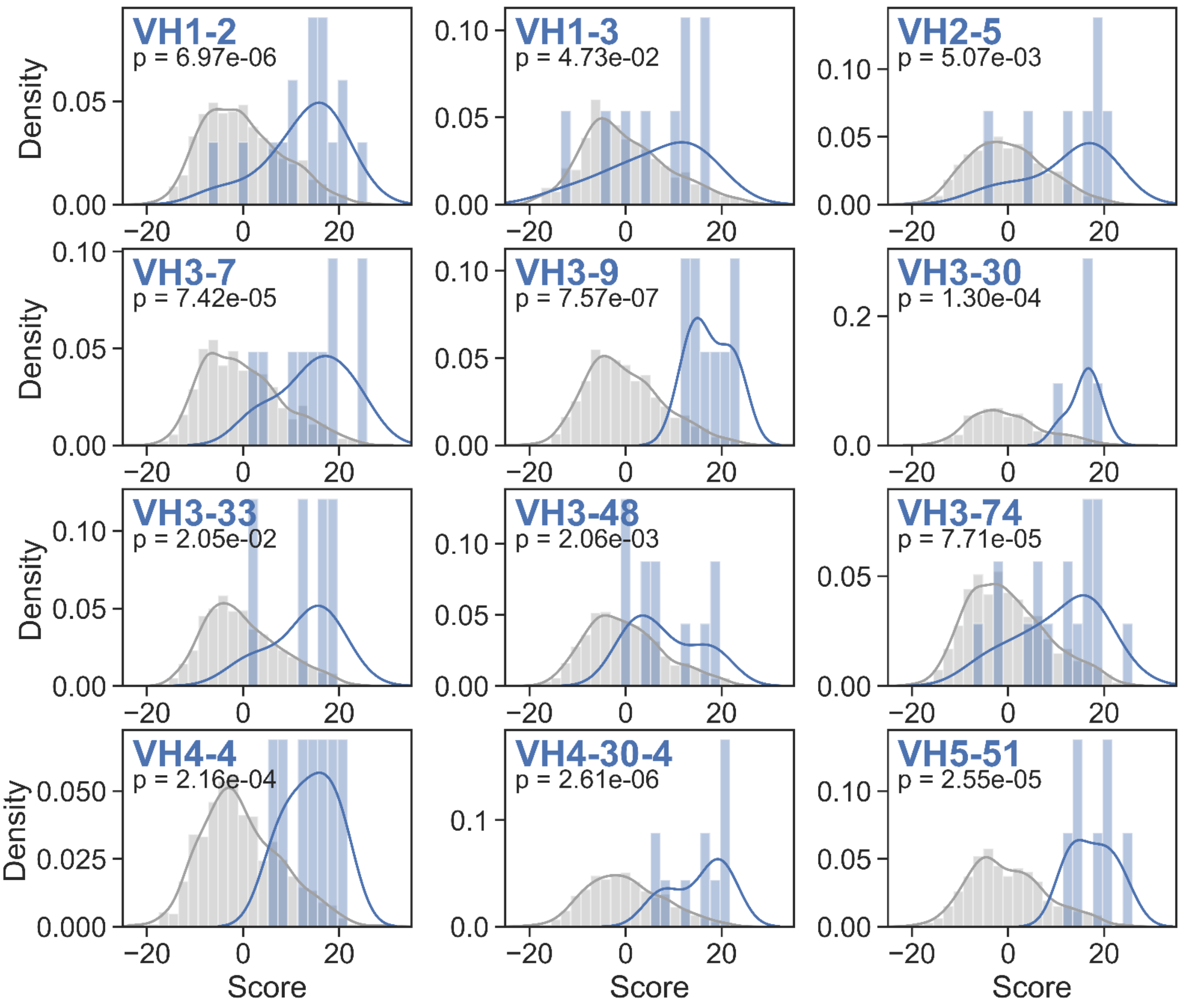

Figure S5 | FDA-approved mutation scores compared to their inferred germline gene PSSMs. Histograms of all germline gene PSSMs generated compared to all FDA-approved framework mutations from their respective germlines. P-values are calculated by one-tailed Welch's t-test.

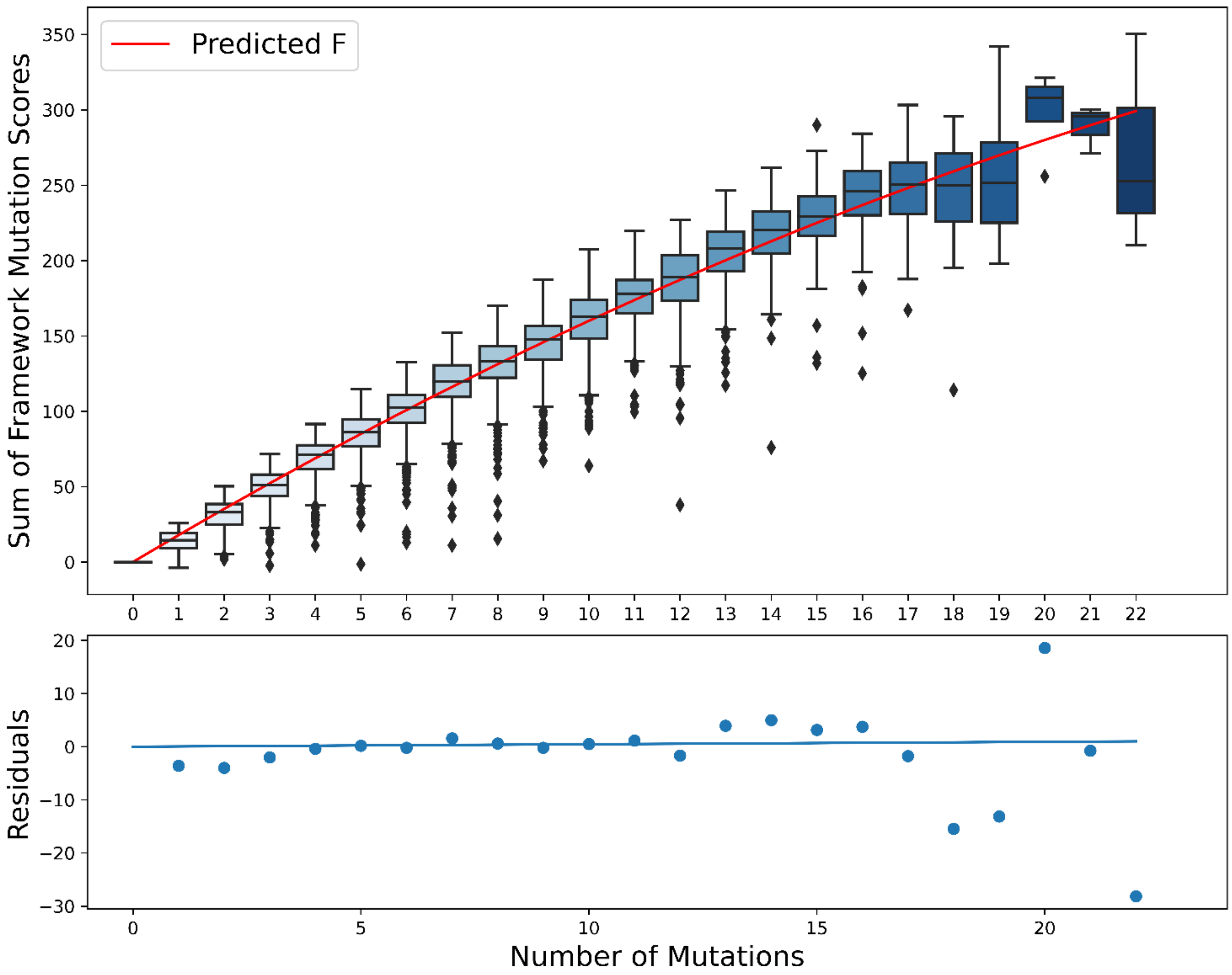

Figure S6 | Least squares regression for normalization. Regression plot for an example germline (VH1-46) for a simple random sample without replacement of 10,000 sequences from the repertoire dataset. Average residuals from fitted line are shown below.

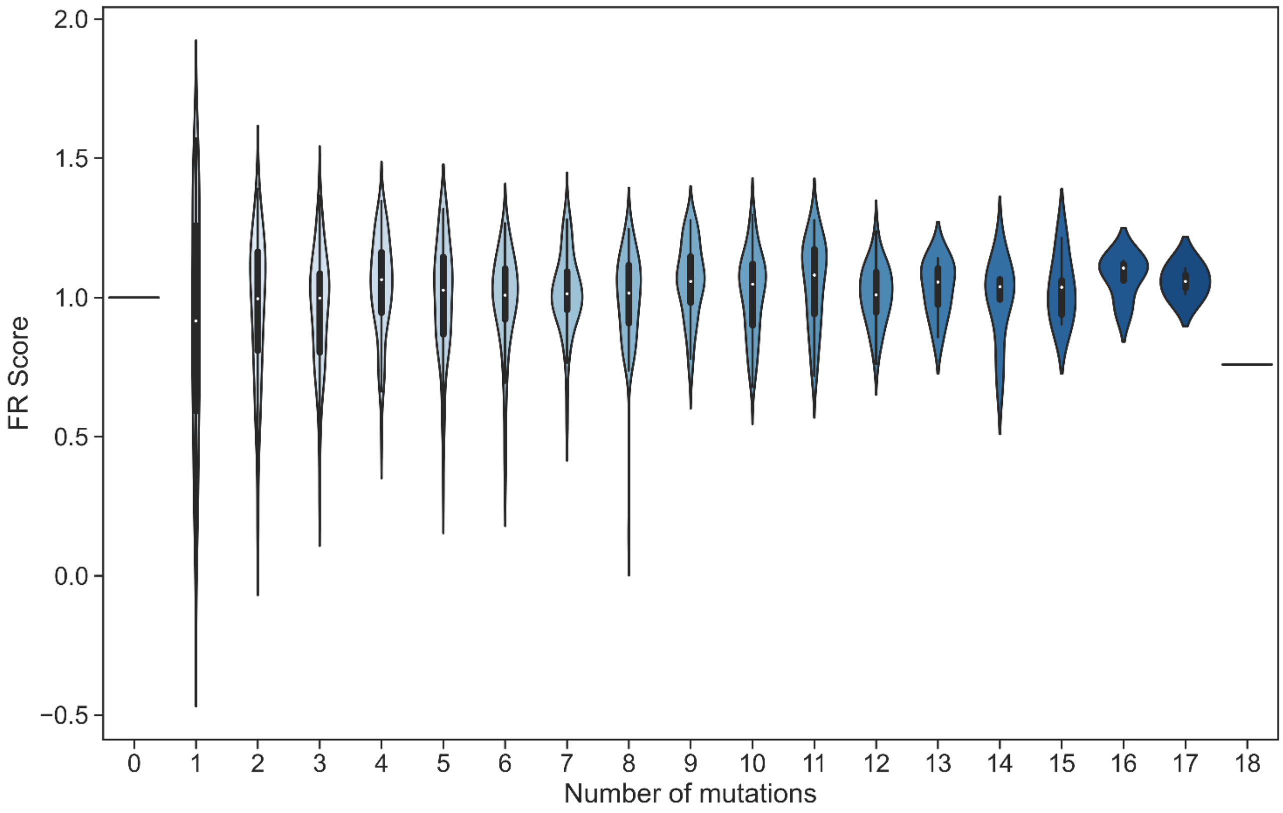

Figure S7 | FR score of randomly sampled repertoire antibodies. FR scores for repertoire antibody sequences were calculated from a simple random sample without replacement of 1,000 sequences across all germlines.

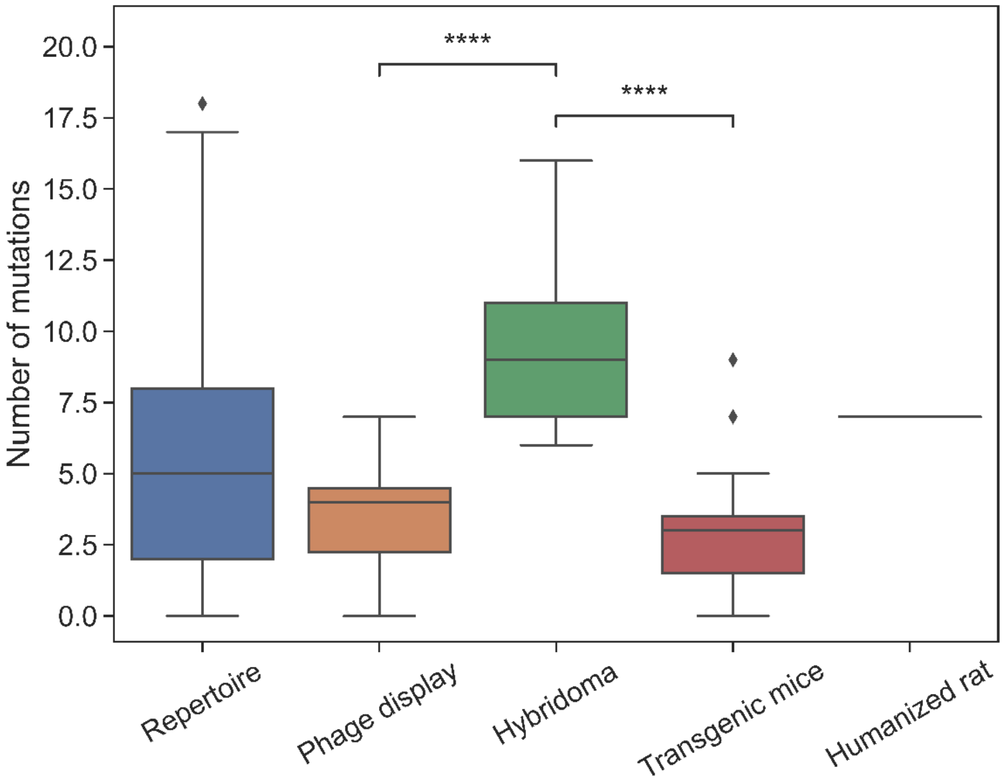

Figure S8 | Regulatory approved antibodies developed by hybridomas have a higher number of framework mutations than those developed by other methods. Number of framework mutations for mAbs is shown by development method. (****: $p < 1e\text{-}4$)

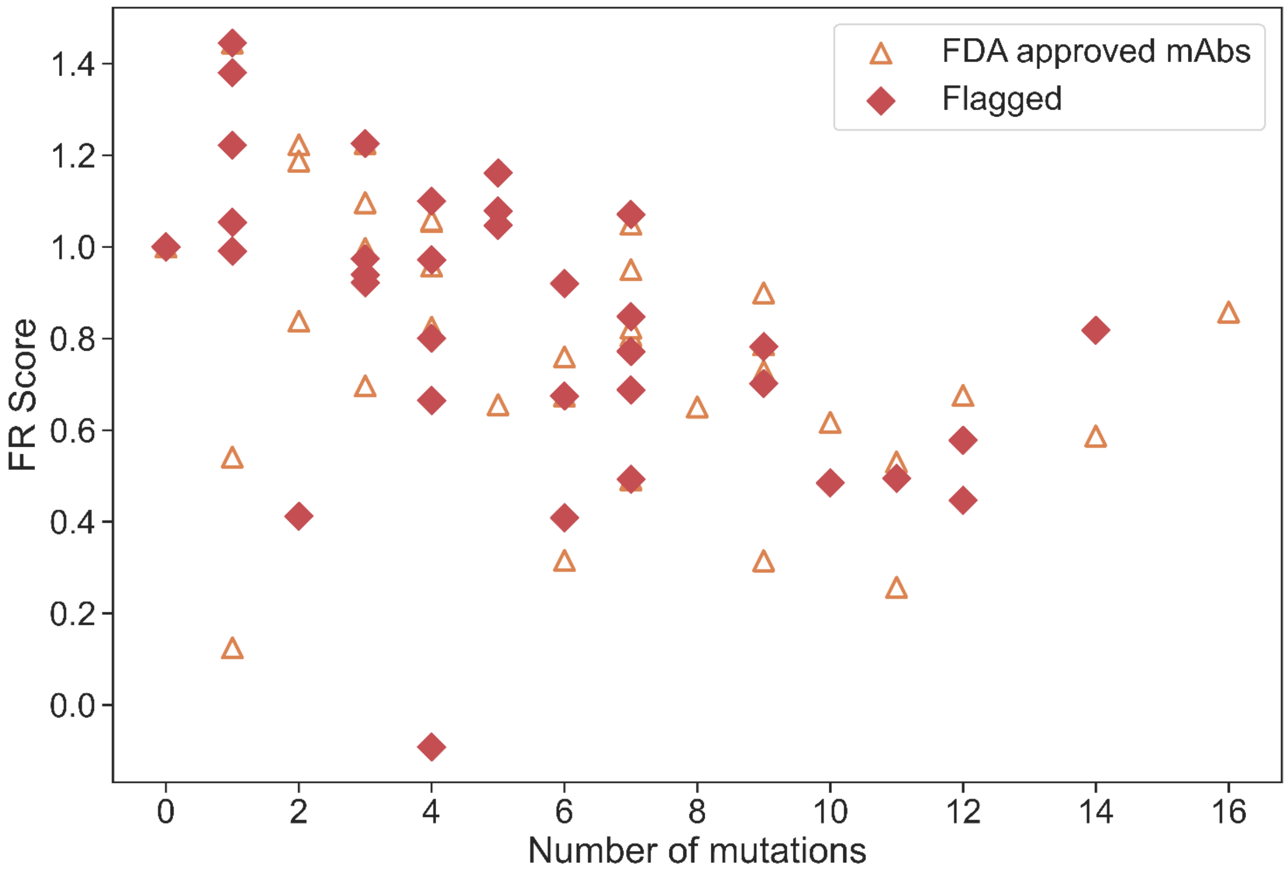

Figure S9 | FR scores of FDA-approved and flagged mAbs versus number of framework mutations.

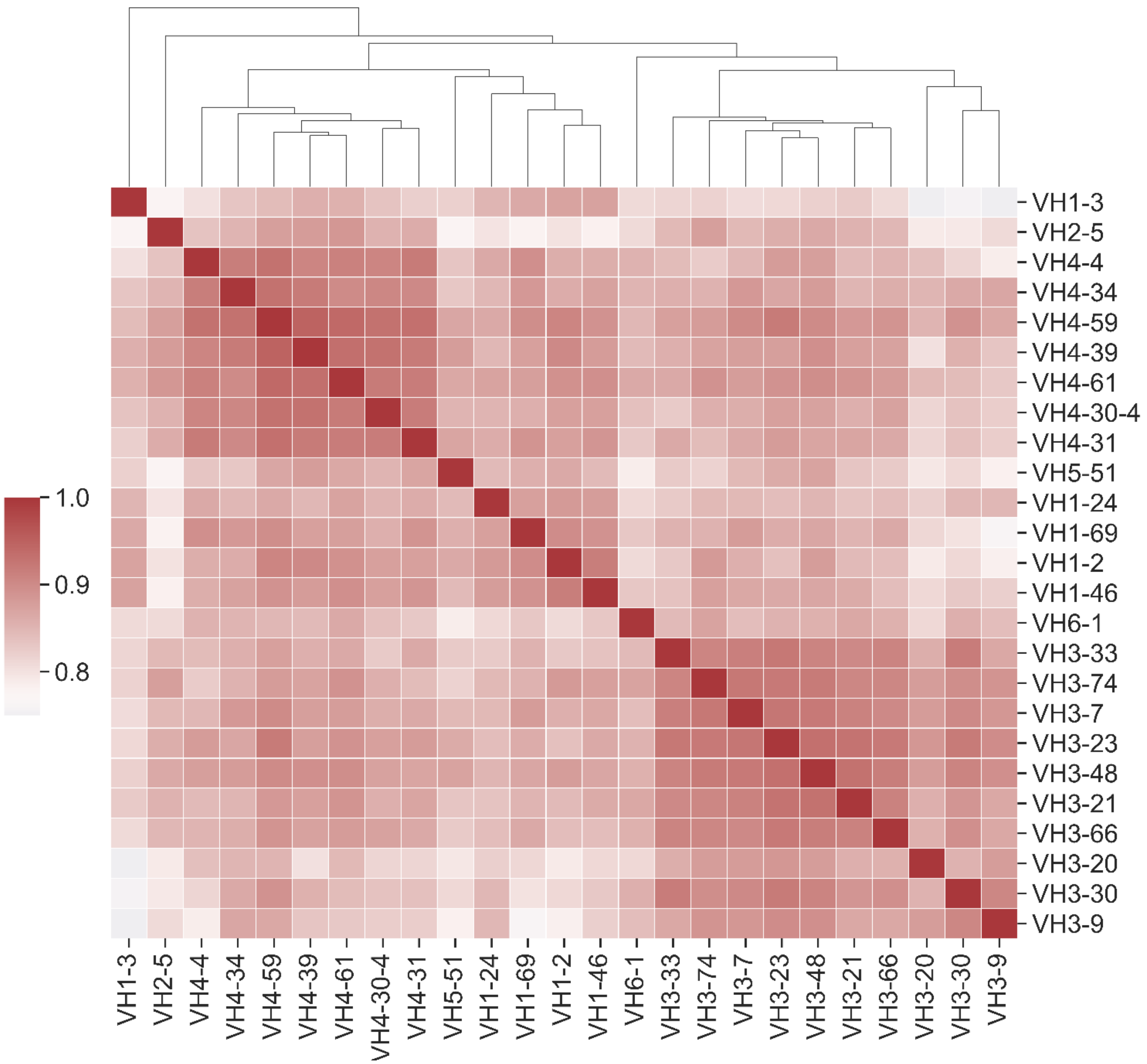

Figure S10 | Framework score correlations between germline VH families for shared germline codons only. Heatmap shows correlations between germline VH families where scores are restricted only to shared codons between any two VH germlines. Germline families are grouped by hierarchical clustering of Pearson correlation coefficients. Dendrogram indicates similarity between germline members.

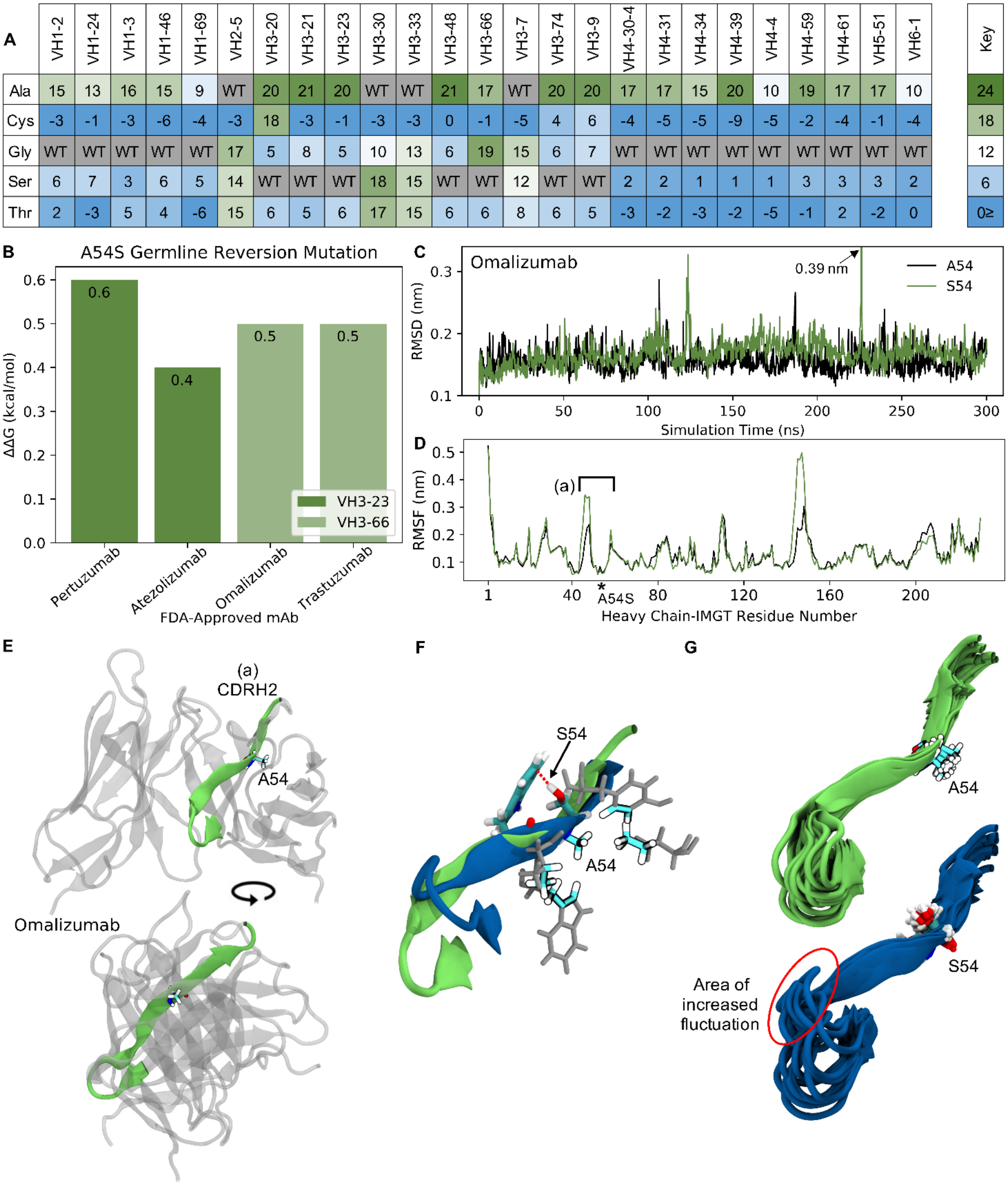

Figure S11 | Molecular dynamics (MD) simulations indicate the 'universal' FR mutation S54A improves mAb stability in VH3-66 germline mAbs. (A) Heatmap of FR scores for the mutation of residue 54 to different amino acids for each of the 25 germline genes. (B) Change in stability as predicted by PremPS upon introducing the A54S germline-reverted mutation into the sequences of four FDA-approved mAbs from two different germlines. (C) Root mean square deviation (RMSD) of Omalizumab with and without the germline-reverted mutation A54S, referenced to the respective energy-minimized structures, as a function of MD simulation time. (D) Root mean square fluctuation

(RMSF) of individual residues in Omalizumab with and without the A54S mutation, calculated from the MD trajectories. (E) Energy-minimized structure of Omalizumab in white, with regions in green indicating residues that experienced relatively large fluctuations in the MD simulations, as indicated in (D) by square brackets. (F) Zoomed-in view of region-of-interest (a) from panels (D) and (E), showing the favorable hydrophobic binding pocket of A54 in Omalizumab (secondary structure in green), versus in the mutant simulation (secondary structure in blue), where S54 rotates to the other side of the beta sheet to find a hydrogen bonding partner (red dashed line). (G) Overlaid simulation snapshots (15, equally spaced across the entire trajectory), highlighting increased fluctuations of loops near the S54 mutation in the mutant simulation (bottom) versus in the mature mAb simulation (top).

2.2 Supplementary Tables

Table S1 | List of all human V_H genes and the subset for which a FR PSSM was generated. Exclusion criteria are listed for germlines for which a PSSM was not generated and sequence counts are listed for created PSSMs.

Germline Gene	PSSM Generated	Exclusion Criteria	Sequences analyzed
VH1-2	Yes	N/A	300,000
VH1-3	Yes	N/A	129,913
VH1-8	No	No associated mAbs	N/A
VH1-18	No	No associated mAbs	N/A
VH1-24	Yes	N/A	260,282
VH1-38-4	No	No associated mAbs	N/A
VH1-45	No	Insufficient number of sequences (2,500)	N/A
VH1-46	Yes	N/A	451,920
VH1-58	No	No associated mAbs	N/A
VH1-68	No	No associated mAbs	N/A
VH1-69	Yes	N/A	300,000
VH2-5	Yes	N/A	141,372
VH2-26	No	No associated mAbs	N/A
VH2-70	No	Insufficient number of sequences (24,100)	N/A
VH3-7	Yes	N/A	2,003,693
VH3-9	Yes	N/A	403,612
VH3-11	No	No associated mAbs	N/A
VH3-13	No	No associated mAbs	N/A
VH3-15	No	No associated mAbs	N/A
VH3-16	No	No associated mAbs	N/A
VH3-19	No	No associated mAbs	N/A

VH3-20	Yes	N/A	175,018
VH3-21	No	No framework mutations	N/A
VH3-22	No	No associated mAbs	N/A
VH3-23	Yes	N/A	300,000
VH3-25	No	No associated mAbs	N/A
VH3-29	No	No associated mAbs	N/A
VH3-30	Yes	N/A	300,000
VH3-32	No	No associated mAbs	N/A
VH3-33	Yes	N/A	710,148
VH3-35	No	No associated mAbs	N/A
VH3-38	No	No associated mAbs	N/A
VH3-41	No	No associated mAbs	N/A
VH3-43	No	No associated mAbs	N/A
VH3-47	No	No associated mAbs	N/A
VH3-48	Yes	N/A	300,000
VH3-49	No	No associated mAbs	N/A
VH3-52	No	No associated mAbs	N/A
VH3-53	No	No associated mAbs	N/A
VH3-62	No	No associated mAbs	N/A
VH3-63	No	No associated mAbs	N/A
VH3-64	No	No associated mAbs	N/A
VH3-66	Yes	N/A	321,361
VH3-69-1	No	No associated mAbs	N/A
VH3-71	No	No associated mAbs	N/A
VH3-72	No	No associated mAbs	N/A
VH3-73	No	No associated mAbs	N/A

VH3-74	Yes	N/A	1,355,540
VH3-NL1	No	No associated mAbs	N/A
VH4-4	Yes	N/A	300,000
VH4-28	No	No associated mAbs	N/A
VH4-30-4	Yes	N/A	160,757
VH4-31	Yes	N/A	373,163
VH4-34	Yes	N/A	300,000
VH4-38	No	No associated mAbs	N/A
VH4-39	Yes	N/A	300,000
VH4-55	No	No associated mAbs	N/A
VH4-59	Yes	N/A	300,000
VH4-61	Yes	N/A	280,777
VH5-10-1	No	No associated mAbs	N/A
VH5-51	Yes	N/A	702,327
VH6-1	Yes	N/A	323,097
VH7-4-1	No	Insufficient number of sequences (74,500)	N/A
VH7-34	No	No associated mAbs	N/A
VH7-81	No	No associated mAbs	N/A
VH8-51-1	No	No associated mAbs	N/A

Table S2 | Sequence identity of IgG sequences from Great Repertoire Project by VH gene.

	IgG	
Gene	% >98% nt identity	% unmutated
VH1-2	22.9	5.6
VH1-3	31	2.1
VH1-24	25	7
VH1-46	25.1	1.7
VH1-69	40.2	0.6
VH2-5	18.3	2.3
VH3-7	9.1	1.5
VH3-9	15.2	1.6
VH3-20	13.2	0.6
VH3-21	22	5.8
VH3-23	12.6	3.1
VH3-30	16.3	1.5
VH3-33	18.8	1.2
VH3-48	13	1.9
VH3-66	14.3	3.5
VH3-74	6.6	0.9
VH4-4	11.3	2
VH4-30-4	24.5	2.2
VH4-31	23.9	2.5
VH4-34	33.7	10.1
VH4-39	14	2.8
VH4-59	17.2	4
VH4-61	19	3.7
VH5-51	23.6	6.4
VH6-1	18.2	3.6
Overall	17.4	3.3

Table S3 | Sequence identity of IgG and IgM sequences from Great Repertoire Project by Subject.

	IgG		IgM	
Patient	% >98% nt identity	% unmutated	% >98% nt identity	% unmutated
316188	7.4	1.5	40.9	10.2
326650	30.2	7.8	75.0	25.1
326737	26.0	5.0	67.6	16.3
326780	15.2	2.2	36.0	6.0
326797	25.9	4.5	55.1	11.8
326907	15.6	3.5	32.7	11.9
327059	11.0	2.0	56.9	16.9
D103	18.7	3.6	66.8	17.7
Overall	17.4	3.3	54.6	14.0

Table S4 | Allelic variations in V_H frameworks for the analyzed germline genes.

Germline Gene/Allele	Allelic Variation(s)	Allele
VH1-2*02	W55R	VH1-2*01, VH1-2*05, VH1-2*06
	Q69H	VH1-2*07
	R75W	VH1-2*04
	M78S	VH1-2*01
	A100V	VH1-2*01, VH1-2*05
VH1-3*01	K70E	VH1-3*02, VH1-3*03
	T99M	VH1-3*02, VH1-3*03
VH1-46*01	F71L	VH1-46*04
VH1-69*13	G55R	VH1-69*02, VH1-69*04, VH1-69*07, VH1-69*08, VH1-69*09, VH1-69*11, VH1-69*15, VH1-69*18
	A80T	VH1-69*05, VH1-69*16
	E82K	VH1-69*02, VH1-69*04, VH1-69*06, VH1-69*08, VH1-69*09, VH1-69*10, VH1-69*14, VH1-69*17
	E97D	VH1-69*03
VH2-5*02	G40S	VH2-5*08
	S68G	VH2-5*05, VH2-5*06, VH2-5*09
	A100G	VH2-5*04
VH3-9*01	T99M	VH3-9*03
VH3-30*18	V55F	VH3-30*02
	A68T	VH3-30*10
	T77A	VH3-30*09
	T86R	VH3-30*13

	N92S<br>D98G	VH3-30*15<br>VH3-30*05
VH3-33*01	H40Y<br>V71A<br>K84T<br>Y88F	VH3-33*07<br>VH3-33*02<br>VH3-33*02<br>VH3-33*02
VH3-48*01	A96D	VH3-48*02
VH3-74*01	S66T	VH3-74*03
VH4-4*07	T24A	VH4-4*01, VH4-4*02, VH4-4*03, VH4-4*04, VH4-4*05, VH4-4*06
	V25I	VH4-4*04
	I42V	VH4-4*01, VH4-4*02, VH4-4*03, VH4-4*04, VH4-4*05, VH4-4*06
	A46P	VH4-4*01, VH4-4*02, VH4-4*03, VH4-4*04, VH4-4*05, VH4-4*06, VH4-4*08, VH4-4*09
	R55E	VH4-4*01, VH4-4*02, VH4-4*03, VH4-4*04, VH4-4*05, VH4-4*06
	R55Y	VH4-4*08, VH4-4*09
	M78I	VH4-4*01, VH4-4*02, VH4-4*03, VH4-4*04, VH4-4*05, VH4-4*08, VH4-4*09

	T82K	VH4-4*01, VH4-4*02, VH4-4*03, VH4-4*04, VH4-4*05
	C103Y	VH4-4*01
VH4-30-4*02	T24A	VH4-30-4*07
VH5-51*01	I39T	VH5-51*02
	R43H	VH5-51*07
	G47R	VH5-51*05
	S83P	VH5-51*04

Table S5 | Normalization constants for FR score.

Constants from least squares regression to fit equation of the form:

$$\sum_{l=0}^{m} S_{ijk} = c_0 m + c_1 m^2$$

where m is the number of framework mutations from germline.

Gene	c_0	c_1	Reduced Chi-Sqr
VH1-2	18.0	-0.20	0.98
VH1-3	20.6	-0.07	0.97
VH1-24	15.0	0	0.98
VH1-46	21.5	-0.36	0.96
VH1-69	14.9	-0.04	0.98
VH2-5	16.6	-0.19	0.97
VH3-7	18.6	-0.19	0.98
VH3-9	19.1	-0.30	0.97
VH3-20	21.4	-0.43	0.98
VH3-21	16.6	-0.12	0.97
VH3-23	17.5	-0.16	0.97
VH3-30	14.2	-0.07	0.97
VH3-33	16.0	-0.03	0.97
VH3-48	17.4	-0.19	0.97
VH3-66	16.0	-0.04	0.97
VH3-74	18.6	-0.27	0.98

VH4-4	18.1	-0.16	0.96
VH4-30-4	16.5	-0.18	0.97
VH4-31	21.5	-0.31	0.98
VH4-34	16.0	-0.05	0.98
VH4-39	17.0	-0.15	0.97
VH4-59	17.6	-0.17	0.98
VH4-61	18.5	-0.25	0.97
VH5-51	18.1	-0.19	0.98
VH6-1	22.2	-0.81	0.93

Table S6 | FR scores and structural coordinates for FDA-approved antibodies.

*2 or more nucleotides needed to obtain substitution (from germline and all known alleles)

FDA Approved Antibody	Germline Gene	Framework Mutations	Mutation Scores	FR Score	PDB ID	Structure Type	Development Method
Pembrolizumab	VH1-2	H40Y	14.5	0.86	5JXE	Fab complex	Hybridoma
		R/W55G	5.3				
		Y67F	18.1				
		A68N	-6.7*				
		Q/H69E	17.4				
		Q72K	14.4				
		G74N	-0.5*				
		S/M78L	20.4				
		R80T	15.5				
		T82S	10.1				
		I84T	17.1				
		S85T	20.7				
		S92K	9.4*				
		R93S	24.5				
		R95Q	8.0*				
		S96F	14.8				
Vedolizumab	VH1-3	A25G	16.8	0.31	Unavailable	N/A	Hybridoma
		M53I	11.4				
		W55E	-12.6*				
		K66N	12.3				
		S68N	-3.4*				
		Q72K	3.5				

		I78L<br>R80V<br>T82I	16.8<br>0.7*<br>11.1				
Benralizumab	VH1-46	M39I<br>A45R<br>E51A<br>I55Y<br>S66K<br>A68N<br>Q69E<br>K70R<br>Q72K<br>R75K<br>M78I<br>R80S<br>T82R<br>Y103L	27.8<br>-0.6*<br>8.2<br>-5.6*<br>11.7*<br>-6.4*<br>13.7<br>22.3<br>11.7<br>5.3<br>13.8<br>18.5<br>8.4<br>6.4*	0.59	Unavailable	N/A	Hybridoma
Ravulizumab	VH1-46	M39I<br>H40Q<br>I55E<br>S66E<br>A68T<br>Q69E<br>K70N<br>Q72K<br>G74D	27.8<br>13.6<br>1.1*<br>4.7*<br>16.7<br>13.7<br>20.0<br>11.7<br>19.8	0.79	Unavailable	N/A	Hybridoma

Burosumab	VH1-46	Y67N	11.4	0.54	Unavailable	N/A	Transgenic mice
Risankizumab	VH1-69	S40H<br>V42M<br>M53I<br>G/R55Y<br>N66K<br>A68N<br>Q69E<br>K70N<br>Q72K<br>R75K	11.3*<br>9.0<br>11.3<br>-5.6*<br>18.1<br>-7.0*<br>14.0<br>18.1<br>10.9<br>9.2	0.67	Unavailable	N/A	Hybridoma
Ixekizumab	VH1-69	S40H<br>G/R55V<br>N66D<br>A68N<br>K70R<br>Q72K	11.3*<br>7.5<br>18.2<br>-7.0*<br>18.2<br>10.9	0.67	6NOV<br>6NOU	Fab<br>scFv	Phage display
Galcanezumab	VH1-69	I39M<br>S40Q<br>G/R55A<br>N66V<br>A68I<br>Q72A<br>G74D	13.1<br>2.2*<br>10.4<br>0.4*<br>3.1*<br>1.7*<br>19.4	0.49	Unavailable	N/A	Hybridoma
Palivizumab	VH2-5	L55D	-3.8*	0.81	Unavailable	N/A	Hybridoma

		R66D<br>S/G68N<br>T77S<br>T90K<br>M91V<br>V97A	4.1*<br>19.9<br>18.1<br>13.4<br>16.0<br>18.4				
Secukinumab	VH3-7	S40N<br>N55A<br>D69G<br>A96V	23.9<br>3.2*<br>16.8<br>24.4	0.96	Unavailable	N/A	Transgenic mice
Fremanezumab	VH3-7	M39I<br>N55E<br>Y66H<br>V68A<br>D69E<br>S70A	12.8<br>2.9*<br>18.8<br>19.4<br>14.7<br>11.0	0.76	Unavailable	N/A	Hybridoma
Durvalumab	VH3-7	None	No Scores	1.00	5X8M<br>5XJ4	Fab complex<br>scFv	Transgenic mice
Adalimumab	VH3-9	G55A<br>G66D<br>K72E<br>L101V	15.5<br>23.6<br>13.3<br>23.1	1.06	4NYL<br>3WD5	Fab<br>Fab complex	Phage display
Ofatumumab	VH3-9	G55T<br>N85K	11.5*<br>19.5*	0.84	3GIZ	Fab	Transgenic mice
Sarilumab	VH3-9	K84E<br>Y88F	15.2<br>21.3	0.95	Unavailable	N/A	Transgenic mice

		S93G	15.2				
Ramucirumab	VH3-21	None	No Scores	1.00	Unavailable	N/A	Phage display
Pertuzumab	VH3-23	S40D<br>S54A<br>A55D<br>Y66I<br>A68N<br>D69Q<br>S70R<br>V71F<br>I78L<br>R80V<br>N82R	6.1*<br>20.3<br>4.6<br>2.7*<br>-8.7<br>1.6*<br>1.5*<br>-0.7*<br>13.3<br>-1.3*<br>5.0*	0.26	4LLU<br>1S78	Fab<br>Fab complex	Hybridoma
Denosumab	VH3-23	A55G	25.1	1.45	Unavailable	N/A	Transgenic mice
Daratumumab	VH3-23	A25V<br>Y103F	20.0<br>22.0	1.23	7DUN<br>7DUO	Fab<br>Fab complex	Transgenic mice
Avelumab	VH3-23	S40M<br>A55S<br>Y66F<br>S70T	4.3*<br>22.8<br>20.7<br>7.8	0.82	5GRJ	Fab complex	Phage display
Dupilumab	VH3-23	A25G<br>S40T<br>A55S	16.9<br>22.8<br>22.8	1.23	6WGB	Fab	Transgenic mice
Emicizumab	VH3-23	M39I<br>S40Q	13.8<br>0.9*	0.32	Unavailable	N/A	Hybridoma

		A55S<br>A68R<br>D69R<br>S70E	22.8<br>2.2<br>-2.4*<br>-6.0*				
Lanadelumab	VH3-23	S40M<br>A55G<br>Y66V	4.3*<br>25.1<br>6.2*	0.70	Unavailable	N/A	Phage display
Atezolizumab	VH3-23	M39I<br>S40H<br>S54A<br>A55W<br>R80A<br>N82T<br>L87A	13.8<br>11.4*<br>20.3<br>-8.3<br>1.2*<br>14.8<br>3.3*	0.49	5X8L	Fab complex	Phage display
Ipilimumab	VH3-30	A54T<br>V101I	16.8<br>16.6*	1.19	6JC2	Fab	Transgenic mice
Erenumab	VH3-30	Y67S<br>A68V<br>Y88F	11.0<br>15.5<br>19.5	1.10	6UMI	Fab	Transgenic mice
Canakinumab	VH3-33	H40N<br>V55I<br>S93G	13.3<br>18.3<br>16.0	0.99	4G5Z	Fab	Transgenic mice
Nivolumab	VH3-33	A24K	2.0*	0.12	5GGQ	Fab	Transgenic mice
Certolizumab pegol	VH3-48	V53M<br>S54G<br>Y55W	1.0*<br>6.2*<br>4.9*	0.53	5WUV	Fab	Hybridoma

		Y66I	3.2*				
		I78F	6.6				
		R80L	0.1*				
		N82T	12.3				
		A83S	17.2				
		N85S	18.8				
		S86T	18.4				
		L87A	0.6*				
Omalizumab	VH3-66	A25V	20.7	0.73	4X7S	Fab	Hybridoma
		M39S	2.9*				
		S40W	-7.2*				
		V42I	15.9				
		S54A	17.1				
		V55S	10.4*				
		Y66N	11.1				
		N82D	17.1				
		L87F	15.0*				
Trastuzumab	VH3-66	M39I	15.2	0.65	6B9Z	Fab	Hybridoma
		S40H	11.1*				
		S54A	17.1				
		V55R	6.0*				
		Y66R	10.9*				
		R80A	-1.7*				
		N82T	17.0				
		L87A	5.9*				

Eptinezumab	VH3-66	A25V<br>S40N<br>S54G<br>D69S<br>S70W<br>V71A<br>N85T<br>L87V<br>Y103F	20.7<br>20.9<br>19.0*<br>1.2*<br>-5.5*<br>16.3<br>8.8<br>23.0<br>22.3	0.90	Unavailable	N/A	Transgenic mice
Efalizumab	VH3-74	H40N<br>V51E<br>R80V<br>N82K<br>A83S	18.3<br>18.3<br>-3.0*<br>5.4<br>17.4	0.66	3EO9	Fab	Transgenic mice
Elotuzumab	VH3-74	H40S<br>V51E<br>V53I<br>S54G<br>R55E<br>S66N<br>D69P<br>V71L<br>G74D<br>R75K<br>T77I<br>T86S	12.1*<br>18.3<br>15.7<br>6.0*<br>3.4*<br>25.0<br>-7.0*<br>12.0<br>8.2<br>-1.7*<br>15.5<br>17.2	0.68	Unavailable	N/A	Hybridoma

Tocilizumab	VH4-30-4	I42V<br>K48R<br>Y66T<br>I78M<br>S79L<br>V80R<br>K90R	18.3<br>16.7<br>7.2*<br>20.6<br>11.6<br>6.1*<br>20.9	0.95	Unavailable	N/A	Hybridoma
Necitumumab	VH4-30-4	Y66D<br>I78M<br>L91V<br>S92N	8.2<br>20.6<br>17.0<br>21.0	1.06	6B3S	Fab complex	Phage display
Alemtuzumab	VH4-4	K48R<br>E55F<br>N66E<br>L71V<br>S74G<br>S79L<br>K90R	16.3<br>6.0<br>8.8*<br>14.7<br>19.6<br>11.7<br>20.7	0.82	Unavailable	N/A	Humanized rat
Ustekinumab	VH5-51	I39L<br>E51D<br>M53I<br>I78M<br>A80V<br>S85T<br>S92N	11.9<br>13.9<br>13.5<br>17.7<br>24.6<br>21.5<br>20.2	1.05	3HMW	Fab	Transgenic mice
Guselkumab	VH5-51	None	No Scores	1.00	Unavailable	N/A	Phage display

Table S7 | FR scores for flagged antibodies.

Flagged Antibodies	Germline Gene	Number of Mutations	FR score
Bimagrumab	VH1-2	4	0.80
Emibetuzumab	VH1-2	7	0.85
Blosozumab	VH1-24	4	-0.09
Lenzilumab	VH1-3	1	1.38
Bococizumab	VH1-46	6	0.41
Codrituzumab	VH1-46	9	0.78
Imgatuzumab	VH1-46	7	0.77
Ixekizumab	VH1-46	6	0.67
Ozanezumab	VH1-46	10	0.48
Ponzeumab	VH1-46	6	0.92
Simtuzumab	VH1-46	14	0.82
Visilizumab	VH1-46	12	0.58
Belimumab	VH1-69	7	1.07
Cixutumumab	VH1-69	0	1.00
Lirilumab	VH1-69	0	1.00
Drozitumab	VH3-20	3	0.97
Atezolizumab	VH3-23	7	0.49
Denosumab	VH3-23	1	1.45
Dupilumab	VH3-23	3	1.23
Figitumumab	VH3-23	5	1.08
Gantenerumab	VH3-23	0	1.00
Seribantumab	VH3-23	3	0.94
Sirukumab	VH3-23	7	0.69
Briakinumab	VH3-30	0	1.00
Golimumab	VH3-30	2	0.41

Eldelumab	VH3-33	5	1.16
Foralumab	VH3-33	1	1.05
Tremelimumab	VH3-33	0	1.00
Robatumumab	VH3-48	3	0.92
Etrolizumab	VH3-66	11	0.49
Duligotuzumab	VH3-74	9	0.70
Parsatuzumab	VH3-74	12	0.45
Glembatumumab	VH4-31	4	0.97
Patritumab	VH4-34	1	0.99
Urelumab	VH4-34	4	1.10
Olaratumab	VH4-39	5	1.05
Rilotumumab	VH4-59	1	1.22
Dalotuzumab	VH4-61	4	0.66
Guselkumab	VH5-51	0	1

Table S8 | Heatmap of scores across all analyzed germline genes for “universal” FR mutations.

	VH1-2	VH1-24	VH1-3	VH1-46	VH1-69	VH2-5	VH3-20	VH3-21	VH3-23	VH3-30	VH3-33	VH3-48	VH3-66	VH3-7	VH3-74	VH3-9	VH4-30-4	VH4-31	VH4-34	VH4-39	VH4-4	VH4-59	VH4-61	VH5-51	VH6-1
42L	17	15	20	14	15	14	9	11	12	9	10	11	8	14	12	11	16	19	15	17	15	18	20	13	16
48R	14	15	18	16	13	19	17	18	17	16	15	17	18	19	14	17	17	20	18	18	16	18	17	17	30
53L	17	18	22	16	19	#WT	17	14	13	18	16	21	13	18	14	15	16	17	14	16	14	16	14	17	31
54A	15	13	16	15	9	#WT	20	21	20	#WT	#WT	21	17	#WT	20	20	17	17	15	20	10	19	17	17	9
72E	13	13	10	10	15	18	13	13	13	13	13	15	10	17	15	13	19	20	17	19	18	18	20	20	14
72R	16	17	14	17	15	22	22	23	21	19	21	22	21	22	20	21	20	21	17	20	19	20	20	17	26
76L	14	19	18	19	19	#WT	10	14	14	12	12	12	12	12	14	10	22	25	18	19	19	19	17	7	15
77A	14	12	14	14	11	13	15	14	16	#WT	14	14	18	13	11	13	18	19	14	16	19	16	15	11	15
77I	13	16	14	13	8	10	14	13	15	14	15	13	17	15	15	15	17	19	17	14	15	15	12	15	17
77S	17	17	17	17	17	19	15	17	17	16	17	16	18	19	19	16	20	23	19	19	17	19	18	17	17
78L	20	19	17	21	17	14	11	10	13	11	10	11	13	11	10	12	15	19	18	18	16	18	14	16	10
78V	16	17	20	26	13	19	16	20	19	19	19	20	18	18	22	19	15	15	16	18	15	15	13	13	25
85S	#WT	14	#WT	#WT	#WT	17	14	18	20	17	17	19	14	18	17	17	16	19	18	17	16	19	16	#WT	11
87V	21	16	24	#WT	23	#WT	17	22	22	21	24	21	23	21	25	19	12	13	16	16	18	18	15	17	14
88F	15	15	24	16	15	13	20	23	22	20	#WT	22	21	23	22	21	13	13	10	11	15	15	14	17	6
92T	20	20	20	16	19	#WT	13	15	14	14	14	15	13	14	14	14	23	25	24	24	26	25	24	18	15
101I	20	17	18	20	19	10	12	20	22	17	18	20	16	21	19	15	15	19	18	18	20	20	18	25	15
103F	21	21	19	22	20	22	20	21	22	19	19	20	22	22	21	21	21	22	21	21	19	21	20	20	18
